# Validation of a triglyceride meter for use in pregnancy

**DOI:** 10.1186/1756-0500-7-679

**Published:** 2014-09-29

**Authors:** Helen L Barrett, Marloes Dekker Nitert, Michael D’Emden, H David McIntyre, Leonie K Callaway

**Affiliations:** UQ Centre for Clinical Research, The University of Queensland, Herston, Queensland Australia; Obstetric Medicine, Royal Brisbane and Women’s Hospital, Herston, Queensland Australia; Endocrinology, Royal Brisbane and Women’s Hospital, Herston, Queensland Australia; School of Medicine, The University of Queensland, St Lucia, Queensland Australia; Mater Medical Research Institute, Queensland, Australia

**Keywords:** Triglycerides, Pregnancy, Diabetes, Point of care

## Abstract

**Background:**

Elevated maternal triglycerides have been associated with adverse pregnancy outcomes including an increased risk of preeclampsia and macrosomia. A valid triglyceride meter would allow the examination of maternal postprandial triglycerides in a systematic manner. A non-fasting venous and two capillary measurements (using the Roche Accutrend® Plus meter) of triglycerides were measured in 40 participants at a mean of 36 weeks gestation.

**Findings:**

The two methods were highly correlated (r = 0.89, P <0.0001), and the distributions were similar (mean difference 0.01 mmol/L (SD 0.47)), t = 0.18, P =0.86). Passing Bablok equation was: y = −0.01 + 0.98 × [95% CI intercept −0.51 – 0. 38; 95% CI slope 0.85-1.15). The estimated bias was −0.01 mmol/L (95% CI −0.93 – 0.91)).

**Conclusions:**

This study demonstrated the Accutrend® Plus meter provides results that correlate strongly with the reference method, with low bias, when used in late pregnancy.

## Background

In pregnancies complicated by diabetes mellitus as well as in non-diabetic pregnancy, elevated maternal triglycerides are associated with adverse pregnancy outcomes including an increased risk of preeclampsia [1], and macrosomia [2–4]. Most studies have examined fasting maternal triglycerides, or random triglycerides either at a single time point or at isolated times across pregnancy. In comparison, regular home based measurement of postprandial glucose is a cornerstone of the management of diabetes during pregnancy. Elevated postprandial triglycerides are associated with adverse outcomes in the non-pregnant population [5] but this has not been systematically studied in pregnancy. Examination of the role of maternal postprandial triglycerides in pregnancy outcomes has been hampered by the lack of an appropriately accurate, practical and validated method.

In late pregnancy, maternal metabolism undergoes multiple alterations including increased triglyceride present in very low density lipoprotein, high density lipoprotein and low density lipoprotein [6, 7]. The alterations in lipoprotein composition during late pregnancy could affect the performance of point of care assays. The purpose of this study was to validate the Roche Accutrend® Plus system using triglyceride strips, in pregnancy.

## Methods

40 women were enrolled in late pregnancy. Gestational diabetes mellitus (GDM) was diagnosed according to the Australasian Diabetes in Pregnancy Society criteria [8]. Random morning venous serum triglycerides (Beckman DXC800) were performed, with simultaneous dual measurement of capillary triglycerides using the Accutrend® Plus system (Roche Diagnostics, Mannheim, Germany). The Beckman DXC800 measures triglycerides by cleavage of the triglycerides into glycerol and free fatty acids, then further enzymatic steps producing a dye, and this dye product is measured by change in absorbance at 520 nanometers. Our clinical laboratory is a commercial laboratory, National Association of Testing Authorities, Australia (NATA) accredited to ISO 15189 standard. The Accutrend® Plus test works by cleavage of the triglycerides into glycerol and free fatty acid, with further enzymatic steps producing hydrogen peroxide, the concentration of which is measured by reflectance photometry. The Accutrend® Plus manufacturer reports within series imprecision CV of up to 3.4% (in the pathological range) and day to day imprecision CV 2.3% and measurement range of 0.80 – 6.86 mmol/L. The capillary triglycerides were measured using the same meter for each woman, but with variable meters used between women. The venous sample was transported on ice to the laboratory and the triglycerides measured in the general clinical laboratory run on the day of sampling.

Normality of data was assessed using Shapiro-Wilk tests. The accuracy of the Accutrend® Plus was determined by comparing the results of the simultaneously sampled venous plasma and the first of the two capillary results measured, using paired sample t-tests and Pearson’s correlation coefficients. Data is presented as mean and standard deviation unless otherwise noted. The agreement between capillary and reference method was assessed using Passing Bablok regression [9] and Bland-Altman analysis [10]. These analyses were also performed for the mean of the capillary measurements compared with the reference method and the results were similar (not shown). Sensitivity analyses were performed excluding the six women with normal glucose tolerance and this did not alter the results. Statistical analyses were performed using MedCalc Statistical Software version 14.8.1 (MedCalc Software bvba, Ostend, Belgium; http://www.medcalc.org; 2014).

Permission for the study was granted by The Royal Brisbane and Women’s Hospital Human Research Ethics Committee and The University of Queensland Human Research Ethics Committee. All women gave written informed consent. The study was funded by the Research Advisory Committee, The Royal Brisbane and Women’ s Hospital.

## Findings

Venous and concomitant capillary samples were collected from 40 women at an average gestational age of 253 (SD 5) days. Thirty-four of the 40 subjects had been diagnosed and were being treated for gestational diabetes mellitus; the remaining 6 had normal glucose tolerance. Three of the women with gestational diabetes mellitus were being managed with insulin therapy, two with metformin and one with both insulin and metformin. Maternal characteristics were as follows: mean age 31.0 (SD 9.9) years, mean gestation of delivery 253 (SD 5) days, recalled pre-pregnancy body mass index (BMI) 26.7 (SD 6.3) kg/m^2^ mean HbA1c for the women with gestational diabetes mellitus 5.44% (SD 0.47) (36 mmol/mol (SD 5.1)), mean HbA1c for normoglycaemic women 5.22% (SD 0.16) (34 mmol/mol (SD 1.7)), mean hematocrit 0.38% (SD 0.03) and mean serum creatinine 48.4 μmol/L (SD 7.8).

**Figure 1:**
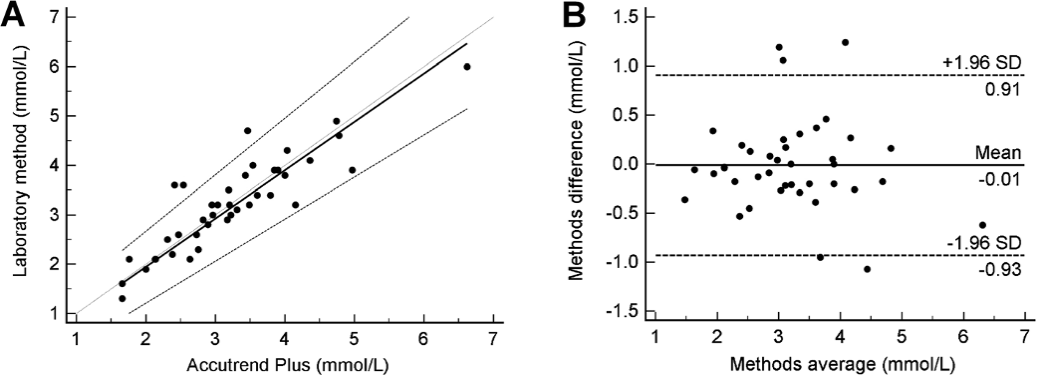
**Graphs of comparison between methods. A**. Passing Bablok regression plot for triglycerides determined on laboratory method and Accutrend® Plus. **B**. Bland Altman Plot for the difference between methods.

The mean maternal serum triglyceride was 3.21 mmol/L (SD 0.97). The reference method was highly correlated with the capillary method (r = 0.89, P <0.0001), and the two distributions were not statistically different (mean difference 0.01 mmol/L (SD 0.47), t = 0.18, P =0.86). Passing Bablok regression equation was: y = − 0.01 + 0.98 × (95% CI for the intercept −0.51 – 0.38; 95% CI for the slope 0.85 – 1.15) (Figure 1A). The estimated bias was −0.01 mmol/L (−0.5%) (95% CI −0.93 – 0.91 mmol/L (−28.5% - 27.5%)) (Figure 1B). The 95% CI of the bias was broad. There was no significant difference between the two capillary triglyceride measurements (3.23 mmol/L (SD 1.01); 3.13 mmol/L (SD1.02): mean difference −0.09 mmol/L (SD 0.71) t = −0.81; P = 0.42)).

## Discussion

This is the first study to examine the measurement of maternal triglycerides in late pregnancy using the Roche Accutrend® Plus. The Accutrend® Plus has previously been examined in two studies in the non-pregnant population. Both studies reported acceptable agreement between methods [11, 12]. The mean bias found in the current study (−0.01 mmol/L (−0.5%)) was smaller than that found in either of the previous two studies using the Accutrend® Plus (8.8% [11]; 0.26 mmol/L [12]). While the mean bias measured in the current study was low, the 95% confidence intervals of the bias was broad but not dis-similar to those found in previous studies of this meter [11, 12]. This may be contributed to by person to person variability in the difference between methods or by the formal venous triglycerides being measured in the clinical run rather than in a single batch. Performing the formal triglycerides in the clinical run may have introduced more error than otherwise would have been found but is more reflective of a potential “real use” situation.

A concern raised by Scafoglieri [12] was that the Accutrend® Plus should not be used for diagnosis of hypertriglyceridemia. Given this, in addition to method comparison, it would be useful to undertake practicability analyses. These have yet to be performed for the Accutrend® Plus in pregnancy. In the current study, the correlation between the two capillary measures taken by study staff was high.

It needs to be borne in mind that the purpose of measuring triglyceride levels in the pregnancy setting is not for the diagnosis of maternal hypertriglyceridemia but to potentially allow assessment of the variation in triglycerides throughout the day. This would provide for a detailed assessment of the association of postprandial triglycerides with pregnancy outcomes. Further, the triglyceride meter could be utilized for self-monitoring of postprandial triglycerides as glucose meters are currently used in diabetes in pregnancy, providing feedback on the effects of food choices. Given we have demonstrated that this meter has low bias, it would be possible to use this device in the research setting to examine maternal triglycerides in pregnancy. In the non-pregnancy setting, the Accutrend® GCT meter has been used for home monitoring [13, 14] and was found to be useful.

The primary limitation of the current study is that it has not assessed the ease of use of the meter. In comparison to glucose meters which have been specifically designed for use by the non-clinician, the Accutrend® Plus meter has been designed more as a point of care meter for use in clinical settings. Prior to using the meter on a broader scale, the practicality and reliability of the non-clinician using the meter at home need to be assessed.

## Conclusions

The Accutrend® Plus meter offers a measurement of maternal triglycerides that has low bias when compared with the reference method. While the 95% confidence intervals around the bias are broad, the use of this device in clinical studies of pregnancy, outside a diagnostic setting would seem reasonable. The practicality of use of the meter in the pregnancy setting requires further investigation.
